# “If you prick us, do we not bleed?” Antisemitism and psychosocial health among Jews in Germany

**DOI:** 10.3389/fpsyg.2024.1499295

**Published:** 2025-01-07

**Authors:** Maor Shani, Dana Goldberg, Maarten H. W. van Zalk

**Affiliations:** Department of Developmental Psychology, Institute for Psychology, Osnabrück University, Osnabrück, Germany

**Keywords:** antisemitism, perceived discrimination, racism-related stress, Jewish identity, microaggressions

## Abstract

**Introduction:**

Amid escalating global antisemitism, particularly following the Hamas attack on Israel on October 7, 2023, this study addresses critical gaps in understanding the psychosocial impact of antisemitism on Jewish communities worldwide.

**Methods:**

Focusing on the Jewish community in Germany, we conducted a cross-sectional survey of 420 Jewish individuals (mean age = 40.71 years, SD = 15.90; 57% female). Participants completed measures assessing four distinct forms of perceived and experienced antisemitism: everyday discrimination, microaggressions (subtle antisemitism and collective experiences such as encountering antisemitic comments on social media), vigilance against antisemitism, and perceived prevalence of antisemitism. Psychosocial outcomes—including depression, anxiety, subjective well-being, and social participation—were also measured. Data were analyzed using correlation analyses and multiple linear regressions, and Latent Profile Analysis (LPA) identified distinct groups based on shared perceptions and experiences of antisemitism and levels of Jewish identification.

**Results:**

Results indicate that experiences of antisemitism, particularly everyday discriminatory acts, were significantly associated with poorer mental health outcomes and reduced social participation. The LPA revealed three distinct groups, with the high-identity, high-antisemitism group (53% of the sample) reporting significantly higher anxiety levels than those with average identification and more rare experience with antisemitism.

**Discussion:**

These findings underscore the pervasive nature of antisemitism and its detrimental effects on the well-being of Jewish individuals. The study highlights the need for targeted interventions to promote resilience within Jewish communities and calls for broader societal efforts to combat antisemitism.

## 1 Introduction

Antisemitic incidents have surged worldwide, increasing by 235% in 2023, particularly in the United States and Europe (Anti-Defamation League, 2024). Following the Hamas attack on Israel on 7 October 2023, the German Association for Research and Information on Antisemitism (RIAS) documented 994 antisemitic incidents in Germany between October 7 and November 9—a 320% increase over the same period in 2022 (Fischer and Wetzels, 2024). These incidents, ranging from violence and property damage to threats and offensive behavior, have eroded the Jewish community’s sense of safety. A 2024 report by the European Union Agency for Fundamental Rights found that 77% of Jewish respondents in Germany viewed public hostility toward Jews as a significant problem, with 52% personally experiencing antisemitism in public spaces (European Union Agency for Fundamental Rights, 2024).

Given this alarming situation, it is crucial to ask: what are the consequences of antisemitism? At the societal level, antisemitism—like other forms of racial discrimination and bigotry—may damage social cohesion, reducing trust and cooperation among community members (Bonick, 2021). In Germany, it hinders reconciliation with the past and the commitment to Holocaust remembrance and nurturing Jewish life (Jikeli, 2020). From a human rights perspective, antisemitism infringes upon basic freedoms, including freedom of religion, equality, and the ability to practice cultural life free from harassment (OHCHR, 2019). Importantly, antisemitism may detrimentally affect victims’ everyday lives, psychological health, and wellbeing. Persistent exposure may lead to chronic stress, anxiety, and depression among Jewish individuals, potentially impacting their overall quality of life (Harrell, 2000; Rosen et al., 2018). The fear of antisemitism can result in behavioral changes, such as concealing one’s Jewish identity or avoiding public spaces, leading to social isolation and reduced participation in community life (Himmelstein et al., 2015; Watson-Singleton et al., 2019). These effects may extend beyond the individual, influencing family dynamics, career choices, and residential decisions, thereby shaping the broader Jewish experience in contemporary society.

Indeed, extensive social psychology research shows that chronic exposure to discrimination among stigmatized groups is linked to maladaptive physiological outcomes like high blood pressure, obesity, and diabetes (Pascoe and Richman, 2009; Lee and Ahn, 2012). It also affects psychosocial health, including depression, anxiety, social interactions, subjective wellbeing (SWB), and self-esteem (Pascoe and Richman, 2009; Schmitt et al., 2014; Paradies et al., 2015; Williams et al., 2019; Priest et al., 2021; Emmer et al., 2024). The theory of racism-related stress posits that racism, whether perceived or real, acts as a chronic psychological stressor, detrimentally impacting both mental and physical health over time (Harrell, 2000; Ong et al., 2009; O’Connor et al., 2021; Ahuja and Haeny, 2024). Racial stigma spans social contexts and can be a source of devaluation (Miller and Kaiser, 2001). Repeated exposure to racism may lead to chronic overactivation of stress pathways, contributing to metabolic issues that impair development and potentially cause early aging, heart disease, and other illnesses (Selvarajah et al., 2022). Notably, even microaggressions—subtle and sometimes unintentional discrimination—can cause chronic stress that negatively affects mental health and quality of life, indicating poor psychological adjustment (Hu and Taylor, 2016; Lui and Quezada, 2019). These effects have been observed across various minority groups, although most research has focused on American minorities like Blacks, Latinos, and Asians.

Despite a growing body of research on the outcomes of racism, relatively little is known about the impact of antisemitism on Jewish communities across the diaspora (Enstad, 2023; Walker et al., 2024). This gap is notable, given that research consistently shows Jews report significantly higher fear of victimization compared to other religious groups (Enstad, 2024; Scheitle et al., 2023). Using data from the EU Fundamental Rights Agency’s 2018 survey, Enstad (2024) found that higher perceived prevalence of societal antisemitism strongly predicts experiences of antisemitic victimization, including harassment and violence. Furthermore, country-level unfavorable opinions of Israel and the presence of larger Muslim populations were identified as predictors of increased experiences and perceptions of antisemitic victimization, supporting the concept of “new antisemitism.”

Several factors contribute to this research gap. First, perceiving Jews as “white” in race, class, and culture has led to their exclusion from core analyses in racism research. This “passing” as white, while sometimes seen as advantageous, paradoxically fuels antisemitic attitudes viewing Jews as part of an undeserved elite (Stein et al., 2024). Second, the misconception of antisemitism as a historical artifact with little contemporary relevance persists, despite the current surge in anti-Jewish rhetoric and hate crimes (Kressel and Kressel, 2016). Third, the complexity and evolving nature of antisemitism across societal segments presents significant methodological challenges for researchers (Salzborn et al., 2011). Lastly, ongoing debates about what constitutes antisemitism, particularly regarding the Israeli–Palestinian conflict and the concept of “New antisemitism,” have politicized the topic, making it contentious even in scholarly fields (Waxman et al., 2022).

We adopted Zick et al.’s (2011) definition of antisemitism as “a social prejudice directed against Jews simply because they are Jewish.” This definition provides a broad socio-psychological lens for examining the associations between perceived or experienced antisemitism and psychosocial health, focusing on individual perceptions and responses. However, we also recognize the importance of definitions that address the specific contemporary manifestations of antisemitism, such as the International Holocaust Remembrance Alliance (IHRA) definition. The IHRA definition delineates explicit examples of antisemitism, including Holocaust denial, conspiracy theories, and anti-Israel rhetoric (Weitzman, 2019). We utilized this discourse-specific IHRA definition to construct measurement items reflecting contemporary expressions of antisemitism (see below).

Perceived antisemitism, adapted from definitions of perceived discrimination (Pascoe and Richman, 2009; Schmitt et al., 2014; Williams and Mohammed, 2009), refers to the subjective experience of being treated unfairly, excluded, or denigrated because one’s Jewish identity. Building on Zick et al.’s (2011) definition, perceived antisemitism encompasses individuals’ interpretations of behaviors, beliefs, or institutional practices that they attribute to antisemitic bias. This definition acknowledges that the psychosocial effects of antisemitism depend on the victim’s perception of discrimination, regardless of the perpetrator’s intent or whether the incident aligns with societal definitions of antisemitism. Consequently, the terms “perceived antisemitism” and “experienced antisemitism” are used interchangeably throughout this article to reflect the subjective nature of these reports. While we situate perceived antisemitism within theoretical frameworks on racial discrimination, we also emphasize that antisemitism possesses unique historical, cultural, and social characteristics, as well as distinctive contemporary expressions, as highlighted in ongoing academic debates (e.g., Cousin and Fine, 2012; Yuval-Davis, 2024). Nevertheless, we believe that established theoretical models linking experiences of racism to psychosocial health outcomes, such as racism-related stress (Harrell, 2000), minority stress theory (Meyer, 2003), and linked lives theory (Elder et al., 2003), offer valuable insights into the effects of antisemitism. These frameworks have been successfully applied across multiple forms of discrimination, including antisemitism (e.g., Rosen et al., 2018; Trilesnik et al., 2022).

Despite limited research, some recent studies have examined the psychological impact of antisemitism. In the United States, direct experiences of antisemitism have been linked to lower life satisfaction, decreased wellbeing, reduced self-esteem, and larger negative affect (Altman, 2011; Chase, 2018; Rosen et al., 2018). Such experiences also strongly predict feelings of being an outsider within one’s community (Alper and Olson, 2011) and correlate with higher levels of depression and a greater propensity for survivor guilt (Kosdon et al., 2021). However, instruments like the Antisemitism-Related Stress Inventory—which combine exposure frequency and stress reactions—make it challenging to isolate the specific impact on wellbeing (Paradies et al., 2015). In Canada, both explicit and ambiguous discrimination have been found to predict increased depressive symptoms among Jewish individuals (Matheson et al., 2019). Similarly, in Australia, concerns about antisemitism and Israel following traumatic events were positively related to increased anxiety, mediating the impact of exposure to graphic and distressing content on social media (Bankier-Karp and Graham, 2024). Although German studies are fewer, they reveal comparable patterns: experiences of antisemitism are associated with heightened worries, fears, and perceived burdens (Zick et al., 2017), significantly limiting individuals’ lives and hindering self-determination (Reimer-Gordinskaya and Tzschiesche, 2021). Among German and Austrian ex-Soviet Jews, perceived antisemitism predicts lower quality of life, while mental health appears more adversely affected by perceived xenophobia than by perceived antisemitism (Trilesnik et al., 2022).

Collectively, these studies show consistent associations between perceived antisemitism and negative mental health outcomes among Jewish individuals—including decreased life satisfaction, increased anxiety and depression, and feelings of social exclusion. However, prior research predominantly focused on blatant, overt discrimination based on ethnic or religious affiliation. To better capture contemporary Jewish experiences with antisemitism in Western societies, it is necessary to address additional domains. Modern antisemitism manifests in complex forms beyond direct discrimination, often appearing as cultural codes, tropes, or metonymical worldviews within political, historical, and cultural contexts (Schuller, 2021). These include conspiracy theories, stereotypes, and delegitimizing beliefs about Israel as a Jewish collectivity (Weitzman, 2019). Moreover, antisemitism frequently appears as subtle microaggressions and collective-discursive forms, especially in online spaces and public discourse (Becker and Bolton, 2022; Weitzman et al., 2023).

These developments mirror the evolution of modern racism and prejudice. Research suggests that ethno-racial prejudice is nowadays more likely expressed subtly or symbolically, as overt expressions are socially unacceptable (Pettigrew and Meertens, 1995; Akrami et al., 2000; Stanke et al., 2024). And still, recent studies indicated that subtle prejudice is no less harmful to minorities’ adjustment outcomes than blatant discrimination (Jones et al., 2016), whether these acts are attributed by targets to racism (Carter and Murphy, 2015; Platow et al., 2022) or cause stress due to their ambiguous interpretation (Cuevas et al., 2024). In this context, racial microaggressions are identified as subtle, indirect expressions of racism (Kanter et al., 2017).

Parallel to such subtle manifestations of antisemitism, Jewish experiences can be affected by vicarious racism—indirect exposure through hearing about or witnessing racism in one’s environment or media (Harrell, 2000; Gee et al., 2012; Himmelstein et al., 2015; Heard-Garris et al., 2018). Social identity theory posits that self-image may be influenced by collective self-esteem, shaped by perceptions of norms and outgroup evaluations (Crocker and Major, 1989; Tajfel and Turner, 2004; Liang and Fassinger, 2008). Actions that devalue or stigmatize the ingroup may negatively affect self-concept and contribute to cumulative racism-related stress on psychosocial health. The theory of linked lives suggests that injustices against ingroup members lead to personal distress (Elder et al., 2003; Gee et al., 2012). Neuroscience evidence indicates that indirect racism can have spillover effects, activating brain regions associated with ostracism, exclusion, and rejection (Masten et al., 2013; Berger and Sarnyai, 2015). These notions suggest that racist or traumatic events can impact health among targeted group members (e.g., Tynes et al., 2019) or even extend beyond the immediate ingroup.

Finally, research on minority stressors has focused on concealing one’s identity to avoid victimization (e.g., Beyer et al., 2024; Meyer, 2003; Lo et al., 2024). Vigilance to racial discrimination involves heightened attentiveness to the environment in anticipation of experiencing racism (Sawyer et al., 2012; Himmelstein et al., 2015). Beyer et al. (2024) recently found that hiding or concealing one’s Jewish identity among German Jews was more common among young individuals and those living in areas with higher perceived Muslim population, while direct experiences with antisemitic harassment was less predictive of such vigilance. While sometimes a coping mechanism, this vigilance can lead to intense worry, persistent monitoring, and social avoidance—the consequences of living “on alert.” During the COVID-19 pandemic, waves of racism against Asian and Black Americans showed that vigilance practices independently contributed to racism-related stress (LaVeist et al., 2014; Chae et al., 2021). In the context of antisemitism, Jews may contend not only with antisemitic experiences but also with the anticipation of such encounters (Reimer-Gordinskaya and Tzschiesche, 2021). This anticipation may lead some to conceal their Jewish identity in public, such as avoiding Jewish symbols or disguising religious practices. Such measures can add to anticipatory stress or perseverative cognitions—chronic worries and fears—which exacerbate the negative effects of racism-related stress on physical and mental health (Sawyer et al., 2012).

In conclusion, given this complexity in contemporary prejudice, it is crucial to examine both overt discriminatory acts and subtle, collective forms of antisemitism to fully understand their psychological impact on Jewish individuals and communities. A comprehensive approach is therefore essential for capturing the unique characteristics of contemporary antisemitism and its potential effects on mental health and social behavior.

### 1.1 The present study

This study aims to fill research gaps on perceived and experienced antisemitism and its outcomes by conducting a cross-sectional analysis among Jews living in Germany. Nearly eight decades after the fall of the Third Reich, the Jewish community in Germany numbers about 100,000 individuals—barely 0.2% of the population—with many immigrants from the former Soviet Union (Geller and Meng, 2020). As antisemitic incidents rise globally, understanding their psychological toll becomes increasingly crucial. This understanding can inform targeted interventions to mitigate negative psychological consequences like anxiety, stress, and social withdrawal (European Union Agency for Fundamental Rights, 2024), raise awareness among policymakers for effective prevention strategies, and provide insights into resilience factors applicable to combating other forms of discrimination. Given Germany’s unique historical context, understanding contemporary antisemitism is essential for addressing the Holocaust’s legacy and ensuring the safety and wellbeing of Jewish communities.

The main objective was to examine relationships between perceptions and experiences of antisemitism and indicators of psychosocial health. In our theoretical review, we emphasized the importance of considering various forms of antisemitic discrimination, as different types of experiences can have distinct psychological impacts (Paradies et al., 2015; Williams et al., 2019; Zick et al., 2017). Traditionally, research has focused on overt, blatant discrimination based on ethnic or religious affiliation, which we operationalized as perceived antisemitic discrimination or *everyday antisemitism*, adapted from “everyday racism” scales (Williams et al., 1997). However, recent literature highlights the significance of subtle forms of discrimination, such as microaggressions, which can be equally harmful to mental health (Jones et al., 2016; Sue, 2010). Based on Sue’s (2010) framework, we distinguished between two types of microaggressions: subtle antisemitism, which are covert, sometimes automatic responses from non-Jews relying on stereotypes and prejudice; and collective antisemitism, involving devaluations and antisemitic expressions communicated publicly without directly targeting individuals, such as exposure to antisemitic hate speech online. These forms capture both direct subtle insults and indirect or public expressions of antisemitism that individuals may encounter. Furthermore, the literature points to the unique effects of vigilance against antisemitism, which involves heightened alertness to discrimination and efforts to reduce the likelihood of being targeted, an experience found to contribute to poor mental health among minorities (Himmelstein et al., 2015). Lastly, we considered the perceived prevalence of antisemitism in society, as research shows that merely perceiving antisemitism as a significant societal issue can impact individuals’ wellbeing, even without direct or indirect experiences (Paradies et al., 2015; Williams et al., 2019; Zick et al., 2017). Each of these types captures different dimensions of antisemitic experiences that may uniquely contribute to psychosocial stress and health outcomes among Jews.

Regarding psychosocial outcomes, we focused on common mental health indicators: depression and anxiety, which are frequently examined in the context of perceived discrimination and linked to negative long-term outcomes and poor quality of life (Schmitt et al., 2014; Priest et al., 2021). We also examined associations between perceived antisemitism and SWB, referring to overall life satisfaction (Diener, 1994). Finally, we explored the manifestation of antisemitism in social life. Social participation, an important aspect of social capital, is linked to both psychological and physiological health (Berkman et al., 2000). Limited research has so far been published on social participation as an outcome of discrimination. Limited research has examined social participation as an outcome of discrimination; among African Americans, discrimination was found to be a significant risk factor for social exclusion, contributing to poor mental health and feelings of isolation (Saasa, 2019).

Our second objective was to identify and characterize different profiles among members of the Jewish community in Germany based on their experiences with antisemitism and their identification as Jews. By moving beyond linear relationships, we aimed to gain deeper insights through homogeneous clusters and data-driven typologies of antisemitism-related perceptions and experiences, revealing non-linear relationships that conventional methods might overlook. To achieve this, we utilized latent profile analysis (LPA), a flexible categorical modeling approach that classifies participants into latent clusters based on patterns across various variables (Spurk et al., 2020). To our knowledge, no prior studies have employed LPA in research on perceived antisemitism.

In exploring profiles based on antisemitic perceptions and experiences, we included a measure of social identification with Jewish religious or ethno-national identity, as it may influence perceptions and experiences of antisemitism and interact with them to determine psychosocial outcomes. Jews may utilize their Jewish identity as a coping mechanism or buffer against the negative impact of such experiences (Branscombe et al., 1999; Schmitt et al., 2014), or they may distance themselves from the social category as a disengagement strategy (Becker and Tausch, 2014; van Veelen et al., 2020). Prior research has found consistent evidence for a positive relationship between Jewish identification and self-reported experiences of antisemitism (e.g., Dubow et al., 2000; Altman, 2011; Rebhun, 2014; Zick et al., 2017; Trilesnik et al., 2022; Enstad, 2024). Individuals with stronger Jewish identity may be more attuned to recognizing and interpreting incidents as antisemitic due to heightened awareness of Jewish issues and history (Friedlander et al., 2010). Higher ingroup identification may increase Jewish visibility, but can also increase resilience, suggesting that a stronger sense of group cohesion and identity confidence can attenuate the sense of fear and worry (Wallengren and Mellgren, 2015; Enstad, 2024). Social identity theory (Tajfel and Turner, 2004) provides a robust framework for understanding this relationship: individuals derive significant parts of their self-concept from group membership, including religious and ethnic identities. For strongly identified Jews, their Jewish identity becomes central to their self-concept, making them more sensitive to potential threats or discrimination against their group (Kessler and Hollbach, 2005) and more vigilant about subtle forms of antisemitism or microaggressions (Sellers and Shelton, 2003). Furthermore, the rejection-identification model suggests that perceiving discrimination can lead to increased in-group identification as a coping mechanism, creating a reciprocal relationship between Jewish identity strength and antisemitism perception (Branscombe et al., 1999).

Together, these research goals have the potential to significantly contribute to social psychology and antisemitism research. By addressing the understudied psychosocial impact of antisemitism amid the recent global surge in incidents, this study aims to fill a crucial gap in the literature. Examining four distinct forms of perceived antisemitism provides a comprehensive framework for understanding how various manifestations affect mental health and social behavior. This approach may inform future studies on subtle and collective forms of prejudice across different minority groups. Investigating the role of Jewish identification in relation to antisemitic experiences and mental health outcomes could offer valuable insights into coping mechanisms and resilience factors applicable to other forms of discrimination. Employing LPA to identify distinct groups within the Jewish community introduces a novel methodology for understanding heterogeneous responses to discrimination. Ultimately, the findings have the potential to inform the development of targeted interventions to promote resilience among Jewish communities and guide broader efforts to combat antisemitism and other forms of discrimination.

## 2 Materials and methods

All measures, data, and analysis files are publicly available at https://osf.io/4f7s5/?view_only=5c91dd5391aa4726b495237194d8a972. The study was approved by the ethics committee of the authors’ institute.

### 2.1 Participants and procedure

Due to the difficulty of obtaining a representative sample of the Jewish community in Germany, we employed a convenient snowball sampling method to recruit participants aged 18 and above who live in Germany and identify as Jewish. Recruitment was conducted through contacts with Jewish and professional organizations, mailing lists, and personal networks. A total of 619 individuals accessed the online survey between December 2022 and March 2023. After excluding 179 participants with over 90% missing data, four who selected “Christianity” as their religion (retaining three who identified as “Atheists”), and 16 who failed an attention check, the final sample comprised 420 participants—sufficient for statistical power (see Supplementary material). After signing a standard consent form, participants completed questionnaires assessing perceptions and experiences of antisemitism, outcome variables, sociodemographic and sociopolitical characteristics, and other measures not reported here.

The average age of participants was 40.71 years (SD = 15.90). Gender distribution was 57% female (*n* = 239), 42% male (*n* = 177), and three participants chose “other.” German nationality was held by 78%, with the remainder mostly having dual German and Israeli citizenship, only Israeli citizenship, or citizenship of another European country. A majority (80%) had a migration background. Approximately half were born in Germany, 27% in the former Soviet Union, 13% in Israel, and 10% in other countries. Regarding education, 10% did not achieve a high-school diploma (Abitur or equivalent), 32% held a high-school diploma, and 43% had academic education—including 18% with a master’s degree and 10% with a doctoral degree. Socioeconomic status (SES) was perceived as “very good” by 14%, “good” by 50%, “medium” by 30%, “bad” by 6%, and “very bad” by one participant. Employment status included 41% full-time employed, 11% part-time employed, 20% students, and 7% retired. Marital status was 41% married, 19% in a relationship, nearly 30% single, 8% divorced, and 1% widowed; 48% had children. Over 60% resided in large cities with over 500,000 inhabitants, and only 7% lived in towns or villages with fewer than 20,000 residents. Most participants (82%) were members of Jewish communities across the country, and 32% frequently participated in community activities.

### 2.2 Measures

All measures not originally in English, or without an existing validated German version, were translated using a forward-backward translation procedure by a member of the research team highly proficient in both English and German. While we did not explicitly differentiate between online and offline experiences in our measures of everyday antisemitism and subtle antisemitism, these scales were designed to capture antisemitic encounters that could occur in any context, including both physical and virtual environments. Acknowledging the significant role of online platforms in the dissemination of antisemitic content (Becker et al., 2024), the collective antisemitism scale specifically includes items referring to online antisemitism, such as exposure to antisemitic comments on the internet or social networks (e.g., “You see antisemitic comments on the internet, e.g., in social networks”).

#### 2.2.1 Everyday antisemitism

To assess perceptions of direct discriminatory experiences, we used the German-translated Everyday Discrimination Scale (Williams et al., 1997). Participants rated nine items (e.g., “You are treated with less courtesy than other people are”; “People act as if they are afraid of you”) on a 5-point scale (1 = *never* to 5 = very *often*), indicating the frequency they felt discriminated against due to their Jewish, Israeli, or religious affiliation. The scale showed high internal consistency (α = 0.89).

#### 2.2.2 Antisemitic microaggressions

Antisemitic microaggressions were measured using a similar 5-point frequency scale. As mentioned earlier, we measured antisemitic microaggressions with two subscales: subtle and collective antisemitism. The collective antisemitism subscale included items referring to demonization and delegitimization of the Jewish state, in accordance with the IHRA definition of antisemitism and its contemporary manifestations (Weitzman, 2019), such as “You hear or read media reports that demonize Israel, the Israeli military, or the Israeli people.” Specifically, we aimed to cover the spectrum of contemporary manifestations of antisemitism in Germany, including Holocaust-related secondary antisemitism and Israel-related antisemitism (Kempf, 2015). Specifically, subtle antisemitism was assessed with five items (e.g., “Someone makes you feel like you’re not part of German society”; “Someone makes you feel like you have to justify yourself for all sorts of things as a Jewish person”), showing good reliability (α = 0.87). Collective antisemitism was measured with seven items (e.g., “You hear or read conspiracy theories involving Jews”), also with high reliability (α = 0.89).

#### 2.2.3 Perceived prevalence of antisemitism

The perceived prevalence of antisemitism in German society was measured using a sliding scale ranging from 1% to 100%. Participants estimated the percentage of people in German society who hold negative views of Jews.

#### 2.2.4 Vigilance against antisemitism

Vigilance against antisemitism was assessed using nine items adapted from Zick et al.’s (2017) instrument measuring antisemitism-related worries, security perceptions, and behaviors. Participants rated their agreement on a 5-point scale (1 = *completely disagree* to 5 = *completely agree*). Sample items include “There are events and places to which I do not go because of antisemitism” and “I would not want my neighbors to know that I am Jewish.” The scale demonstrated good reliability (α = 0.86).

#### 2.2.5 Depression and anxiety

Depression and anxiety were measured using the Hospital Anxiety and Depression Scale (HADS; Petermann, 2011), a widely used instrument assessing psychological distress on a 4-point scale with varying anchors. After removing one item due to insufficient model fit in a confirmatory factor analysis (CFA) (see below), the anxiety subscale comprised five items (e.g., “Worrying thoughts go through my mind,” α = 0.79), and the depression subscale included six items (e.g., “I have lost interest in my appearance,” α = 0.74).

#### 2.2.6 Subjective wellbeing

Subjective wellbeing was assessed using the Satisfaction with Life scale (Diener, 1994). Participants responded to five items (e.g., “In most ways my life is close to my ideal”) on a 7-point scale ranging from 1 (*not true at all*) to 7 (*completely true*), with high reliability (α = 0.87).

#### 2.2.7 Social participation

Social participation was measured with a four-item scale developed for this study. Participants rated how often they visit friends, meet new people, and engage in activities outside the Jewish community on a 5-point scale (1 = *never* to 5 = *very often*). The scale showed acceptable reliability (α = 0.77).

#### 2.2.8 Jewish identification

Jewish identification was measured using four items adapted from Frindte and Dietrich (2017). Participants indicated their agreement on a 6-point scale (1 = *completely disagree* to 6 = *completely agree*) with statements such as “Being Jewish is an important part of who I am.” The scale’s reliability was acceptable (α = 0.72).

#### 2.2.9 Demographics and personal characteristics

Sociodemographic and personal characteristics were collected as control variables for regression analyses and to characterize latent profiles (see below). These included gender, age, highest educational level, migration background and nationality, family status and number of children, SES, religiosity, political orientation (left-right scale), and frequency of participation in Jewish community events and activities. For all multi-item scales, arithmetic means were calculated to represent each construct, with higher scores indicating a greater degree of the respective quality.

## 3 Results

### 3.1 Preliminary analysis

First, we conducted a confirmatory factor analysis (CFA) to validate the conceptual distinction between everyday, subtle, and collective antisemitic experiences at the measurement level. A correlated three-factor model, with four added inter-item residual covariances (between items 1–2 and 8–9 on the everyday antisemitism scale, and items 6–7 and 7–8 on the collective antisemitism scale), yielded an acceptable model fit according to acceptable criteria (Hu and Bentler, 1999), χ^2^ (182) = 432.635, *p* < 0.001, CFI = 0.944, TLI = 0.935, RMSEA = 0.062, 90% CI [0.054, 0.069], SRMR = 0.061.

Second, we examined the factor structure of the HADS using CFA. The initial two-factor model (depression and anxiety) demonstrated insufficient fit, χ^2^ (55) = 193.489, *p* < 0.001, CFI = 0.987, TLI = 0.0972, RMSEA = 0.083, 90% CI [0.071, 0.096], SRMR = 0.057. Modification indices indicated a high cross-loading on item 4 of the anxiety subscale, which we removed. By adding covariances between the residuals of items 2 and 7 in the anxiety subscale and items 6 and 8 in the depression subscale, the model fit improved significantly: χ^2^ (41) = 100.543, *p* < 0.001, CFI = 0.950, TLI = 0.932, RMSEA = 0.062, 90% CI [0.046, 0.077], SRMR = 0.043.

Finally, the vigilance against antisemitism scale showed a good fit to a single-factor structure, as indicated by a principal component analysis (PCA) with an added residual covariance between items 6 and 7: χ^2^ (26) = 52.57, *p* = 0.002, CFI = 0.977, TLI = 0.968, RMSEA = 0.054, 90% CI [0.033, 0.075], SRMR = 0.040.

Missing data at the item level did not exceed 16.7%. Little’s MCAR test indicated that the data were missing completely at random (χ^2^ (10,591) = 10,738.464, *p* = 0.155; therefore, the data were assumed to be missing at random and cases with missing data were omitted per analysis.

### 3.2 Descriptive analysis

**FIGURE 1:**
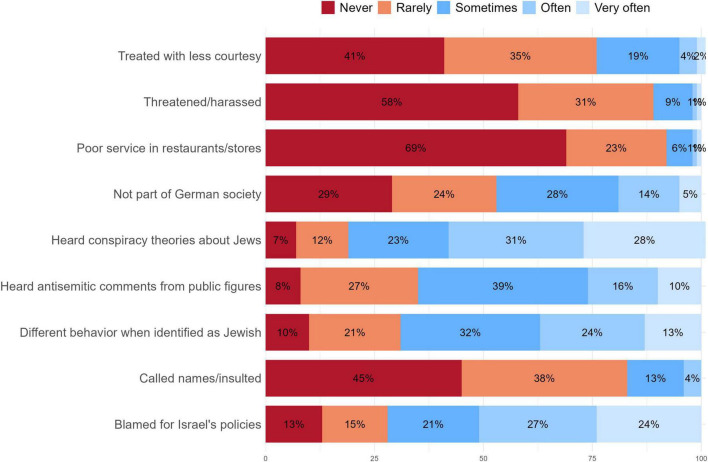
Percentage distribution of selected items measuring everyday, subtle, and collective antisemitism. *N* varies between 412 and 419 due to missing responses across items.

Participants reported experiencing subtle antisemitism more frequently than everyday antisemitism (i.e., perceived direct discrimination). As illustrated in Figure 1, over 40% indicated that they were at least rarely threatened or harassed, and 45% reported being called names or insulted due to their Jewish affiliation. In contrast, more than half of the participants stated they were often or very often blamed for the policies of the State of Israel or exposed to conspiracy theories about Jews. Approximately two-thirds encountered antisemitic statements in the media at least sometimes. Participants in our study estimated that, on average, 45% of Germans harbor antisemitic views (SD = 21.37), a figure significantly higher than recent survey-based estimates. For instance, a 2019 survey by the Anti-Defamation League. (2019) reported that 15% of Germans hold such views. This substantial discrepancy highlights the gap between the perceived prevalence of antisemitism among the German Jews in our sample and broader societal estimates, indicating a heightened perception of antisemitism within the Jewish community.

### 3.3 Correlational analysis

**FIGURE 2:**
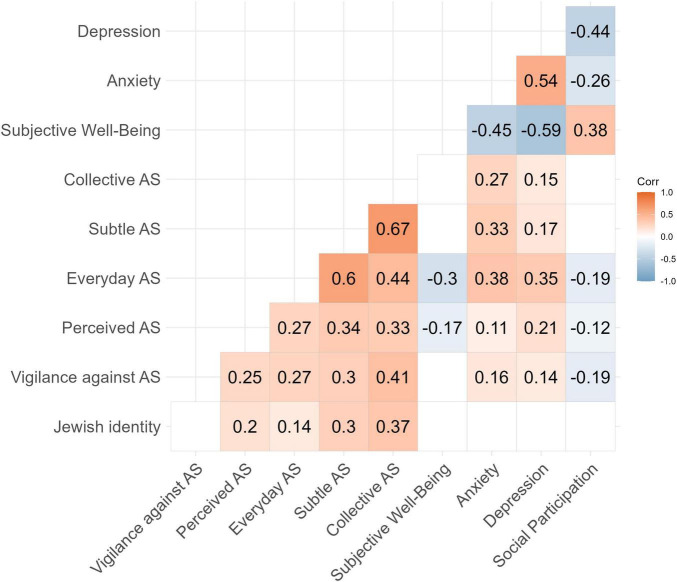
Bivariate Pearson correlations among the study’s main variables. AS, antisemitism. Correlations with absolute values of 0.11 or higher are statistically significant (*p* < 0.05). Non-significant correlations are not displayed (cells remain blank). Stronger colors represent higher correlation coefficients, as indicated by the scale on the right side of the figure.

Pearson correlations among the study’s main variables are presented in Figure 2 (a more conventional correlation matrix is presented in Supplementary Table 1). All measures of perceived everyday, subtle, collective, and prevalent antisemitism were positively correlated. Participants who experienced more everyday antisemitism also tended to report more frequent microaggressions, as measured through subtle and collective antisemitism, perceived a higher prevalence of antisemitism in German society, and, to a lesser extent, exhibited higher vigilance against exposure to antisemitism. More frequent experiences of everyday antisemitism were moderately associated with lower SWB and social participation, as well as higher anxiety and depression. Subtle and collective antisemitism, two dimensions of antisemitic microaggressions, were also moderately linked to increased anxiety and depression. Perceiving a higher prevalence of antisemitism was weakly but significantly related to higher anxiety and depression, and to reduced social participation and SWB. Additionally, embracing more vigilant behavior was weakly to moderately associated with higher anxiety and depression and with lower social participation. Finally, Jewish identification was positively related to all measures of perceived and experienced antisemitism but was not related to vigilance against antisemitism. Interestingly, we found no significant relationship between Jewish identification and psychosocial outcomes.

### 3.4 Multiple linear regressions predicting outcomes from antisemitism

**FIGURE 3:**
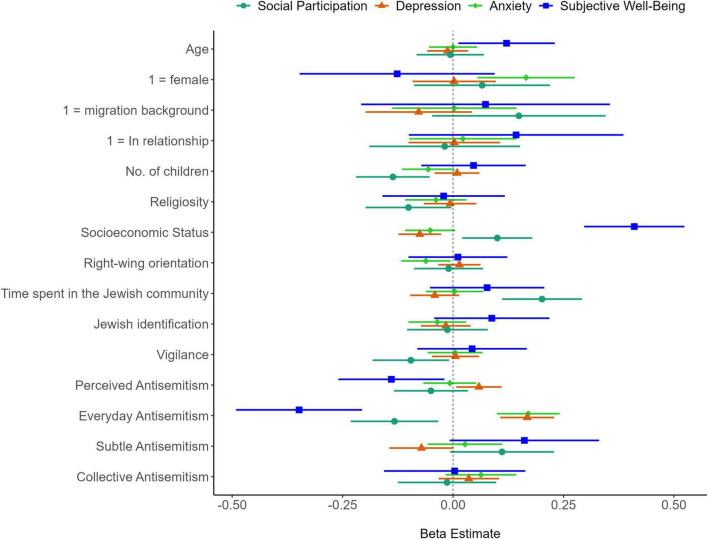
Results of multiple linear regression analyses predicting psychosocial outcomes. The plot presents standardized regression coefficients from four multiple linear regression models, each with 15 predictors displayed on the *y*-axis. These models predict social participation, depression, anxiety, and subjective wellbeing. The horizontal lines extending from each standardized beta estimate represent 95% confidence intervals. Predictors whose confidence intervals do not cross the zero point on the *x*-axis are considered statistically significant (*p* < 0.05).

To examine the unique predictive power of perceived and experienced antisemitism domains beyond demographic variables, we conducted four multiple linear regression analyses on the outcome variables: SWB, anxiety, depression, and social participation. The results are illustrated in Figure 3, with detailed parameters provided in Supplementary Tables 2–5. Regarding demographic predictors, higher age and SES significantly predicted higher SWB. Higher anxiety was associated with being female and leaning toward the left of the political spectrum. Only higher SES significantly predicted lower depression. Higher social participation was predicted by several demographic variables, including having fewer children, higher religiosity, higher SES, and more frequent involvement in the Jewish community.

After controlling for demographics and relevant personal characteristics, everyday antisemitism emerged as a significant predictor of higher anxiety and depression, as well as lower SWB and social participation, surpassing other predictors in the models. Additionally, perceiving a higher prevalence of antisemitism significantly predicted lower SWB and increased depressive symptoms. Vigilance against antisemitism significantly predicted reduced social participation. While the effect of subtle antisemitism on anxiety, depression, and social participation was marginally significant (*p*s < 0.07), collective antisemitism did not uniquely contribute to explaining variance in the outcome variables beyond other forms of antisemitic experiences.

To control for multiple comparisons across the regression models, we applied the Holm-Bonferroni correction to the *p* values of all predictors. The adjusted *p* values for each predictor across the models are presented in Supplementary Table 6. After adjustment, from the main variables, only antisemitic experiences in everyday life remained a consistently significant predictor across all outcomes. In contrast, subtle antisemitism, collective antisemitism, perceived prevalence of antisemitism, vigilance and Jewish identification were not significant in any model after adjustment, with adjusted *p* values above 0.05 across outcomes.

Finally, we conducted an additional series of multiple linear regressions to examine a potential interaction between Jewish identification and everyday antisemitism in predicting the four outcomes (see Supplementary Table 7). The results indicated no significant interactions, suggesting that the strong predictive relationship between everyday antisemitic experiences and psychosocial outcomes is consistent across different levels of identification with the Jewish ethnic or religious identity.

### 3.5 Profiles of experiences with antisemitism and Jewish identification

We conducted a LPA using the R package tidyLPA (Rosenberg et al., 2018). The profiling variables were Jewish identification, vigilance against antisemitism, perceived prevalence of antisemitism, everyday antisemitism, and antisemitic microaggressions (subtle and collective antisemitism). All variables were standardized, and complete cases (*n* = 344) were included. To determine the optimal profile solution, we compared models with different variance and covariance structures: equal variances with fixed covariances (EV/FC), varying variances with fixed covariances (VV/FC), equal variances with equal covariances (EV/EC), and varying variances with varying covariances (VV/VC) (Rosenberg et al., 2018). Models ranging from 1 to 10 profiles were assessed using empirical criteria such as the Bayesian information criterion (BIC), sample size-adjusted BIC (SABIC), and the bootstrapped likelihood ratio test (BLRT) with 999 resamples, which provided *p* values for model improvement (Nylund et al., 2007). Conceptual criteria favored parsimonious models with meaningful profiles representing at least 5% of the sample.

**FIGURE 4:**
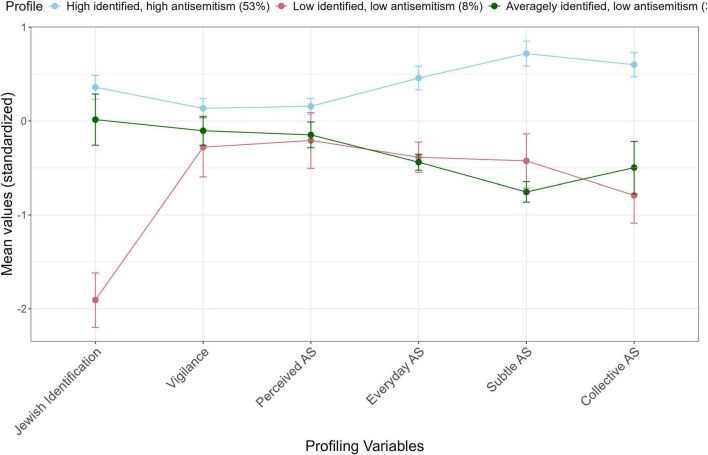
Standardized mean scores (profile centroids) with standard errors for the three-profile solution. AS, antisemitism. Error bars represent one standard error above and below the mean.

The 3-class model with equal variances and equal covariances (EV/EC) was selected as optimal based on low BIC and SABIC values and a significant BLRT (see Supplementary Table 9). Although a 4-class EV/EC model showed slightly better fit, it included a profile representing only 1% of the sample. Clustering means are depicted in Figure 4, and detailed differences are available in Supplementary Table 8. Jewish identification and experiences of everyday, subtle, and collective antisemitism significantly differentiated the profiles. In contrast, vigilance against antisemitism was moderate and similar across profiles, and perceived prevalence of antisemitism was relatively high (over 40% on average) in all profiles.

The first profile, *High Identity*, *High Antisemitism*, comprised 53% of the sample. Individuals in this group (see Table 1) were highly identified with their Jewish identity and experienced antisemitism more frequently than those in other profiles. They were more religious, participated more in Jewish community activities, and 62% were born in Germany.

**TABLE 1:** Demographic information for the three profiles with significance tests for the difference between profiles.

Variable	Profile			Test
	**Profile 1: high identity, high antisemitism (*n* = 181)**	**Profile 2: low identity, low antisemitism (*n* = 29)**	**Profile 3: average identity, low antisemitism (*n* = 134)**	
	***M* (SD)**	***M* (SD)**	***M* (SD)**	**ANOVA/χ^2^**
Age	38.68 (14.55)	35.10 (11.92)	42.23 (17.42)	*F*(2,341) = 3.43, *p* = 0.03, η^2^ = 0.02
Gender	*n* (%)	*n* (%)	*n* (%)	χ^2^ (2) = 1.11, *p* = 0.57
Male	75 (42%)	14 (48%)	51 (38%)	
Female	103 (58%)	15 (52%)	82 (62%)	
Country of birth				χ^2^ (6) = 22.02, *p* = 0.011
Germany	112 (62%)^a,b^	10 (36%)^a^	57 (43%)^b^	
FSU	38 (21%)^a,b^	8 (29%)^a^	46 (34%)^b^	
Israel	14 (8%)^a^	8 (29%)^a,b^	18 (13%)^b^	
Other	16 (9%)	2 (7%)	13 (10%)	
Socio-economic status	3.72 (0.76)	3.46 (0.64)	3.82 (0.83)	*F*(2,338) = 2.54, *p* = 0.08
Political orientation	4.46 (1.86)	4.07 (2.12)	4.42 (1.84)	*F*(2,336) = 0.51, *p* = 0.60
Religiosity	5.12 (2.43)^a^	2.00 (2.41)^a^	3.95 (2.54)^a^	*F*(2,341) = 23.66, *p* < 0.001, η^2^ = 0.12
Time spent in the Jewish community	3.27 (1.15)^a^	2.10 (0.98)^a^	2.87 (1.12)^a^	*F*(2,341) = 15.02, *p* < 0.001, η^2^ = 0.08
Family status				χ^2^ (2) = 1.40, *p* = 0.50
Not in a relationship	68 (38%)	11 (38%)	59 (44%)	
In a relationship	113 (62%)	18 (62%)	75 (56%)	
Number of children	0.97 (1.16)	0.62 (0.82)	0.95 (1.13)	*F*(2,341) = 1.25, *p* = 0.29

*N* = 339–344. Identical row letters denote significant difference between profiles based on Bonferroni-corrected pairwise comparisons (*p* < 0.05). FSU, former Soviet Union.

The second profile, *Low Identity*, *Low Antisemitism*, included 8% of the sample. Participants had significantly lower Jewish identification and reported less perceived and experienced antisemitism compared to profile 1. They were less religious, less active in the Jewish community, and younger on average; 28% were born in Israel, higher than in profiles 1 (8%) and 3 (13%).

The third profile, *Average Identity*, *Low Antisemitism*, accounted for nearly 40% of the sample. These individuals identified less strongly with Jewish identity than those in profile 1 but more than those in profile 2. Their experiences with antisemitism were similar to profile 2. About one-third were immigrants from the former Soviet Union, and they were less religious and less active in the Jewish community compared to profile 1.

We then examined whether the three profiles differed in their average scores on the study’s outcome variables (see Table 2). The groups did not differ significantly in SWB, depression, or social participation. Significant differences emerged only for anxiety, *F*(2,341) = 10.51, *p* < 0.001, η^2^ = 0.07. *Post hoc* Tukey’s HSD tests indicated that high identifiers experiencing frequent antisemitism (profile 1) had higher anxiety levels (*M* = 2.09, SD = 0.55) than those with average identification and low perceived antisemitism (profile 3, *M* = 1.82, SD = 0.53), *p* < 0.001. Interestingly, low identifiers with low perceived antisemitism (profile 2) and high identifiers with high antisemitism (profile 1) had similar levels of anxiety.

**TABLE 2:** Means (standard deviations) and results of one-way ANOVAs for differences between profiles in outcome variables.

	Profile 1: high identity, high antisemitism (*n* = 181)	Profile 2: low identity, low antisemitism (*n* = 29)	Profile 3: average identity, low antisemitism (*n* = 134)	ANOVA	Cohen *d* effect size and significance		
					**Profile 2 vs. profile 1**	**Profile 3 vs. profile 1**	**Profile 3 vs. profile 2**
Subjective wellbeing	4.62 (1.15)	4.22 (1.22)	4.76 (1.14)	*F*(2,341) = 2.67, *p* = 0.07	0.34	0.12	0.47
Anxiety	2.09 (0.55)	2.05 (0.49)	1.82 (0.53)	*F*(2,341) = 10.51, *p* < 0.001	0.07	0.50***	0.44
Depression	1.66 (0.46)	1.66 (0.46)	1.57 (0.46)	*F*(2,341) = 1.70, *p* = 0.19	0.00	0.20	0.20
Social participation	3.16 (0.75(	3.14 (0.71)	3.15 (0.74)	*F*(2,341) = 0.28, *p* = 0.97	0.03	0.01	0.01

Pairwise comparisons were conducted using Tukey’s HSD adjustment.

***p < 0.001.

## 4 Discussion

The study results confirm that antisemitism, in its various forms, continues to be a major stressor for Jews in Germany—a country that has seemingly come to terms with its past and where antisemitism is legally sanctioned. Despite measures to address it, Jewish communities remain vulnerable. Similar to previous studies on exposure to antisemitism among German Jews (European Union Agency for Fundamental Rights, 2024; Zick et al., 2017; Beyer et al., 2024), antisemitism remains a daily reality. Our profiling analysis revealed that more than half of the sample reported high levels of perceived and experienced antisemitism. Subtle forms of antisemitic experiences, such as microaggressions and collective expressions in media and public discourse, were more frequent than overt discriminatory acts, paralleling trends in studies on other minority groups (Stanke et al., 2024).

This research addresses a critical gap at the intersection of antisemitism and the psychological impact of discrimination. Although recent years have seen growing attention to racism’s mental health effects, research on antisemitism’s psychological toll is limited (Altman, 2011; Rosen et al., 2018; Matheson et al., 2019; Kosdon et al., 2021; Trilesnik et al., 2022; Bankier-Karp and Graham, 2024). Our study contributes by showing the potential mental health consequences of antisemitism. While previous studies largely focused on overt discrimination, we examined multiple forms of encounters with antisemitism, including microaggressions (e.g., subtle racism and vicarious experiences), and vigilance. This approach reveals the cumulative and varied effects of perceived antisemitism across contexts. We found that both microaggressions and overt antisemitism were linked to poorer wellbeing and mental health, consistent with findings from research on other groups (Jones et al., 2016). These findings suggest that one need not be a direct victim of severe antisemitic acts for mental health to be affected. Recurrent, mild antisemitic experiences or the belief that antisemitism is widespread can lead to chronic stress, predominantly anxiety. Our regression analysis with correction for multiple comparisons underscored that direct experiences of discrimination were stronger predictors of negative psychosocial outcomes than microaggressions or perceptions of antisemitism’s prevalence in society, aligning with Schmitt et al.’s (2014) meta-analysis on discrimination. However, it would be misleading to assume that contemporary antisemitism is experienced only in subtle forms. Recent research suggests that openly expressed prejudice is becoming socially acceptable again, even in Germany (Crandall et al., 2018; Lees, 2018; Stanke et al., 2024). Blatant antisemitic discrimination is reemerging, indicating that Jews face both subtle and overt forms of discrimination (Fischer and Wetzels, 2024).

Moreover, vigilance against antisemitism—proactive efforts to avoid antisemitic encounters—was associated with reduced social participation, although its predictive power became non-significant when adjusting across regression models. From an exploratory perspective, this finding may be unsurprising, as hypervigilant behaviors, including avoiding social spaces where antisemitic incidents might occur, often lead to isolation (Watson-Singleton et al., 2019; Keum and Li, 2023). Hypervigilance can manifest as constant scanning for antisemitic cues, overinterpretation of ambiguous signals, or heightened arousal in public spaces, prompting withdrawal from social life as a protective measure.

The relationships found in this study between everyday antisemitism and outcomes like SWB, depression, and anxiety appear stronger than those typically reported in meta-analytical studies (Branscombe et al., 1999; Pascoe and Richman, 2009; Emmer et al., 2024), as well as in prior studies on Jews (Altman, 2011; Rosen et al., 2018; Kosdon et al., 2021; Trilesnik et al., 2022). This may reflect the particularly intense nature of antisemitic experiences for German Jews, who may also face other forms of discrimination, such as xenophobia, due to affiliations with groups (e.g., Russians and immigrants), increasing their risk of depressive symptoms (Vargas et al., 2020). Additionally, rising antisemitism and perceptions of inadequate government response may foster frustration and helplessness, amplifying the negative effects of personal experiences (Zick et al., 2017).

Consistent with prior research among American Jews (Friedlander et al., 2010; Altman, 2011; Rebhun, 2014; Chase, 2018) as well as and in European context (Enstad, 2024), our findings show that stronger Jewish identification correlates with more frequent experiences of antisemitism, particularly microaggressions (measured as both subtle and collective forms of discrimination). This is consistent with research showing that stronger identification can increase sensitivity to discriminatory incidents (Molina et al., 2015). Identification also played a key role in distinguishing between profiles of experiences. However, our LPA indicated that while antisemitic experiences varied widely, Jewish identification remained relatively stable across two prominent profiles. In addition, Jewish identification did not significantly predict psychosocial health, contrasting with studies showing positive effects of identity on wellbeing (Smith and Silva, 2011; Cruwys et al., 2015). Accordingly, and despite prior research suggesting that in-group identification can mitigate the negative effects of discrimination (Branscombe et al., 1999; Schmitt et al., 2014), our findings indicate that Jewish identity did not significantly buffer against the harmful psychosocial impacts of perceived antisemitism in this context. This suggests that while identification may influence the perception of antisemitism, it does not necessarily shield individuals from its detrimental mental health effects. However, this discrepancy may also result from measuring identity as an overarching construct or from varying environmental factors, like antisemitism levels, which shape how Jewish ethnic or religious identity protects mental health (Trilesnik et al., 2022). Future studies should explore the nuanced role of Jewish identity in mitigating the negative effects of perceived and experienced antisemitism (Altman, 2011; Trilesnik et al., 2022).

Our LPA revealed three distinct profiles: Slightly more than half of the participants exhibited high Jewish identification and frequent experiences of antisemitism across all forms, characterized by higher religiosity, community involvement, and mostly German-born individuals. About 40% showed moderate identification and relatively low perceived antisemitism, with a notable proportion of immigrants from the former Soviet Union. In contrast, a small minority of participants displayed low identification and infrequent encounters with antisemitism, typically younger, less religious, and with a higher proportion of Israeli-born participants. Although antisemitic experiences were strongly linked to outcomes in regression and correlational analyses, LPA did not reveal significant differences across most outcomes. This discrepancy may be due to differences in analytical methods. Correlational and regression analyses capture linear relationships across the entire sample, while LPA identifies discrete groups, potentially obscuring subtle variations. Extreme antisemitic experiences, as seen in the high-identity, high-antisemitism group, might be linked to significantly worse outcomes (e.g., anxiety), while within-group variability in other profiles could explain the lack of significant differences in SWB or social participation (Scotto Rosato and Baer, 2012). This underscores the importance of examining both identity strength and antisemitic experiences to fully understand their impact on psychosocial health.

Finally, the characterization of the profiles revealed variations in origin-national backgrounds across the groups. The “High Identification, High Antisemitism” profile had a higher proportion of German-born participants (62%) compared to the “Low Identification, Low Antisemitism” (36%) and “Average Identification, Low Antisemitism” (43%) profiles. The “Average Identification, Low Antisemitism” group included a higher proportion of participants from the former Soviet Union (34%), while the “Low Identification, Low Antisemitism” group had a greater representation of Israeli-born participants (29%) compared to the other profiles. These findings suggest that ethno-national origin may play a role in profile membership. It is possible that differences in nationality backgrounds contribute to variations in Jewish identification and perceptions of antisemitism. For example, individuals from the former Soviet Union may have different historical and cultural experiences influencing their identification and perceptions (Dietz, 2003). Similarly, Israeli-born participants might perceive antisemitism differently due to their experiences in a predominantly Jewish society before immigrating to Germany. Intergenerational trauma may also impact these differences. The descendants of Holocaust survivors or those who have familial histories of persecution may have heightened sensitivity to antisemitism and stronger Jewish identification (Klar et al., 2013). These factors could influence both the perception of antisemitism and mental health outcomes. Future research should explore these variables in more depth to understand their influence on Jewish identification, perceptions of antisemitism, and psychosocial health outcomes.

### 4.1 Limitations

Some limitations of this study warrant discussion. First, the cross-sectional design precludes establishing definitive causal relationships between perceived antisemitism and psychosocial health outcomes. While we interpret our findings to suggest that experiences of antisemitism negatively impact mental health, it is also possible that pre-existing mental health conditions or personality traits influence the perception of antisemitism. Individuals with higher levels of anxiety or depression may be more prone to perceive discrimination due to heightened vigilance to threats or negative cognitive biases associated with these conditions (Paradies et al., 2015; Schmitt et al., 2014). Moreover, personality traits such as neuroticism might contribute to both increased perception of discrimination and poorer mental health outcomes (Lewis et al., 2015; Sawyer et al., 2012). While substantial evidence supports the causal effect of perceived discrimination on psychosocial health, including meta-analyses of experimental studies (Schmitt et al., 2014) and longitudinal investigations (e.g., Lavner et al., 2022; Sun et al., 2021), the limited research available on perceived antisemitism prevents us from drawing similar conclusions in this context. Therefore, we acknowledge that the associations observed in our study might be bidirectional or confounded by third variables not accounted for in our analyses. Future research among Jewish communities worldwide should employ longitudinal designs to disentangle the directionality of these relationships and control for potential confounding factors, such as personality traits and baseline mental health conditions.

Another important limitation of our study is that we did not distinguish between different dimensions of Jewish identity when assessing participants’ experiences of discrimination. Jewish identity is multifaceted, encompassing religious, ethnic, cultural, and national aspects, including Israeli identity. Our measures were not designed to disentangle whether discrimination was perceived due to one or more of these facets. Moreover, participants may have experienced discrimination based on other intersecting social identities, such as being immigrants (from Israel, the former Soviet Union, or elsewhere), women, LGBTQ+ individuals, or members of other stigmatized groups. Intersectionality theory posits that individuals can face multiple, overlapping forms of discrimination that interact in complex ways (Gerson, 2018; Lewis et al., 2015). As mentioned above, the intersection of antisemitism with other forms of oppression, such as racism, sexism, and homophobia, may compound the psychological impact on individuals (Smith et al., 2023). Future research should employ an intersectional framework to examine how these multiple identities and forms of discrimination contribute to the experiences and outcomes of Jewish individuals (Trilesnik et al., 2022). Such an approach would allow for a more nuanced understanding of how antisemitism interacts with other forms of oppression and how intersecting identities influence coping mechanisms and mental health outcomes.

An additional limitation of our study pertains to the small size of the “Low Identification, Low Antisemitism” group identified in our LPA, which comprised only 8% of the sample. This small group size may limit the generalizability of findings related to this profile and affect the stability of the results (Nylund et al., 2007). Caution should be exercised when interpreting the characteristics and outcomes associated with this group, and replication with larger samples is recommended.

Furthermore, comparative studies across countries would also be beneficial, considering that experiences with antisemitism may interact with contextual and situational factors. Regarding measurement, all experiences assessed in this study were self-reported perceptions. Future research could incorporate measures less prone to self-report biases, such as direct or indirect observations, or physiological indicators of stress and psychosocial health. Additionally, our sampling technique may have introduced bias: self-identification as Jewish and affiliation with other Jewish individuals or organizations were prerequisites for participation, which implies a certain level of identification. Unaffiliated Jews, whose experiences may differ, were under-sampled. Therefore, caution should be exercised in generalizing these findings to the entire Jewish population in Germany or elsewhere.

### 4.2 Contributions and practical implications

Despite its limitations, this study makes a notable contribution to antisemitism research and the broader field of discrimination studies. By exploring multiple forms of perceived antisemitism, including antisemitic microaggressions, we extend previous research that has predominantly focused on direct discrimination and neglected more frequent subtle and collective experiences, such as comments causing discomfort and online exposure to antisemitic hate speech (e.g., Altman, 2011; Trilesnik et al., 2022). Our findings highlight the pervasive nature of antisemitism in Germany and its detrimental effects on mental health, particularly anxiety and depression, aligning with broader research on racism-related stress (Pascoe and Richman, 2009; Schmitt et al., 2014; Lui and Quezada, 2019; Vargas et al., 2020). Using LPA, we identified distinct groups within the Jewish community, illustrating the nuanced and non-linear relationship between Jewish identification and experiences of antisemitism (Nylund et al., 2007).

Furthermore, this study contributes to the understanding of how perceived antisemitism impacts mental health in modern contexts. It aligns with current trends that underscore the evolving nature of anti-Jewish prejudice, particularly in online spaces where hate speech is often more subtle but no less damaging (Schwarz-Friesel, 2019; Waxman et al., 2022). By integrating psychological research with antisemitism studies, we demonstrated how social psychology can enhance our comprehension of contemporary Jewish life, illustrating the complex relationship between identity, societal attitudes, and individual wellbeing, especially amid rising antisemitism (Anti-Defamation League, 2024; Fischer and Wetzels, 2024).

We view our study and similar studies as a steppingstone toward further research not only on the effects of antisemitism on Jewish individuals and communities but also on the factors that foster resilience and adaptive coping strategies. While research on coping with racism has grown significantly in recent years (e.g., Neville et al., 2024; Holmes et al., 2024), studies focusing on coping mechanisms in the context of antisemitism remain scarce. Beyer et al. (2024), using a mixed-method approach, identified strategies such as withdrawal, intentions of displacement, political advocacy, and collective action to combat antisemitism but did not examine their association with resilient or adaptive outcomes. Similarly, Pearl’s (2023) qualitative study of American Jews highlighted the potential of Jewish identity as a coping mechanism, including benefits derived from community connection, cultural pride, engagement with Jewish traditions, and a historical perspective emphasizing survival as a source of strength. More research is needed to explore engagement- and disengagement-based coping strategies and sources of resilience, particularly as awareness of antisemitism’s harmful impact increases, especially in the aftermath of the October 7 attacks. On a practical level, our findings underscore the urgent need for targeted interventions that address the full spectrum of antisemitism, from overt acts to microaggressions and societal prejudice. While Jewish organizations in Germany currently focus on major hate crime victims (Reimer-Gordinskaya and Tzschiesche, 2021), our results highlight the importance of extending support to those experiencing less overt forms of discrimination. Although research specifically evaluating psychological interventions for coping with antisemitism is limited (Moffic et al., 2020), insights can be drawn from interventions developed for other groups experiencing discrimination.

The link between vigilance against antisemitism and reduced social participation emphasizes the need for safe spaces where Jewish individuals can engage in community life without fear. Strengthening social support networks within Jewish communities could be particularly beneficial, especially since high identifiers experience more antisemitism and are more involved in the community. Community-based programs can foster a sense of belonging and collective identity, which has been shown to buffer against the negative effects of discrimination (Brondolo et al., 2009). Such programs may empower individuals to share experiences, reduce feelings of isolation, and develop collective coping strategies.

Interventions that promote positive ethnic and cultural identity—such as cultural pride reinforcement and engagement with Jewish traditions and history—may also enhance resilience, especially among adolescents and youth (Saleem and Lambert, 2016; Steinhardt, 2022). Engaging in cultural activities and education can reinforce a positive Jewish identity, counteracting the negative impacts of antisemitism. Psychoeducational programs may increase awareness of antisemitism and its effects, equipping individuals with knowledge and strategies to navigate discriminatory environments (e.g., Fleming et al., 2024; Biancalani et al., 2024). These programs may also promote advocacy and empowerment, encouraging individuals to engage in collective action against antisemitism, which fosters a sense of agency and improves psychosocial health (Bartlett et al., 2022; Hope et al., 2018). Future research should focus on developing and rigorously testing evidence-based interventions tailored to the unique experiences of antisemitism. Such research is crucial to inform effective strategies that enhance coping and resilience among Jewish individuals and mitigate the adverse psychological impacts identified in our study. By exploring and implementing these interventions, we can work toward alleviating the psychological burden of antisemitism and strengthening the resilience and wellbeing of Jewish communities.

We believe that our findings have significant implications for a wide range of professionals who provide therapy, counseling, and support to Jewish individuals and communities. This includes social workers, psychologists, counselors, therapists, and other mental health practitioners who play a crucial role in addressing the psychological impact of antisemitism and supporting Jewish clients in developing effective coping strategies. At the micro and mezzo levels, professionals are encouraged to support and be sensitive to the unique needs and concerns of Jewish clients. Practicing cultural humility and self-reflection is essential in examining personal biases and ensuring that services are culturally competent (Gottlieb and Steigerwald, 2024). Mental health practitioners should be aware of the various forms of antisemitism—including microaggressions and societal prejudice—and understand how these experiences can affect mental health and wellbeing. Professionals can assist clients in addressing spiritual and cultural microaggressions by creating a therapeutic environment that respects religious freedom and acknowledges the significance of Jewish identity (Hodge and Boddie, 2021). Supporting clients in reinforcing a positive cultural identity may counteract the negative impacts of antisemitism and contribute to improved mental health outcomes. At the macro level, professionals are encouraged to engage in advocacy and leadership to combat antisemitism and promote social justice. This includes educating themselves and others about antisemitism, challenging antisemitic narratives, and supporting policies that protect the rights and wellbeing of Jewish communities (Gottlieb and Steigerwald). By standing against antisemitism and supporting Jewish individuals and communities, professionals uphold their commitment to human rights and ethical practice.

Finally, our study may also offer valuable insights for policymakers and educators on the widespread nature of antisemitism and its significant psychological toll, highlighting the urgent need for more effective prevention strategies, awareness campaigns, and allocation of resources to increase resilience among Jewish communities (European Union Agency for Fundamental Rights, 2024).

In conclusion, the scarcity of research on the negative outcomes of perceived antisemitism contrasts sharply with its prevalence. Studying its harmful effects is crucial to dismantling the misconception that antisemitism is marginal in Western societies. Our study may contribute to our understanding of how discrimination affects not only Jews but also other minority groups, which may further facilitate efforts toward a more inclusive and equitable society. However, it is important to emphasize that addressing antisemitism cannot be the sole responsibility of its victims, and affected societies should work toward eliminating antisemitism and other forms of prejudice through intensive interventions in all relevant areas of life. It is our hope that by investigating the severity of antisemitic experiences, identifying risk factors, and developing effective coping strategies, we can advance societal efforts to eliminate prejudice and discrimination, supporting both Jews and other minorities in thriving as individuals and communities.

## 5 Conclusion

Antisemitism remains a pervasive aspect of daily life for Jews in Germany, with over half of our sample reporting high levels of perceived and experienced antisemitism. While microaggressions, that is, subtle and collective forms were more frequent, everyday discriminatory acts emerged as the strongest predictors of poor mental health outcomes. Specifically, everyday antisemitism was significantly associated with higher anxiety and depression, and lower SWB and social participation, even after controlling for demographic factors.

Latent profile analysis revealed three distinct groups based on Jewish identification and antisemitic experiences. The largest group (53%) had high Jewish identification and frequent antisemitism experiences, reporting higher anxiety levels. A small group (8%) with low identification and low antisemitism experiences also showed high anxiety, suggesting a complex relationship between identity, antisemitism experiences, and psychological outcomes. The third group (40%) displayed average identification and low perceived antisemitism.

These findings highlight that even mild, recurring antisemitic experiences or perceiving antisemitism as widespread can lead to chronic stress and negative mental health outcomes. Vigilance against antisemitism was associated with reduced social participation, indicating potential social isolation. Collectively, these results emphasize the need for targeted interventions that address various forms of antisemitism and consider the diverse profiles within the Jewish community.

## Data Availability

The datasets presented in this study can be found in online repositories. The names of the repository/repositories and accession number(s) can be found below: https://osf.io/4f7s5/.
