# Supplementary material for: “If you prick us, do we not bleed?” Antisemitism and psychosocial health among Jews in Germany
Source: Front Psychol. 2025 Jan 7;15:1499295. doi: 10.3389/fpsyg.2024.1499295 (PMC11747309; doi:10.3389/fpsyg.2024.1499295)
Supplement: Supplementary file 1 [file Data_Sheet_1.docx]

Supplementary Material

**Title: ‘If You Prick Us, Do We Not Bleed’? Antisemitism and Psychosocial Health Among Jews in Germany**

**Power analysis and sample size requirements**

Monte Carlo power analysis for indirect effects using a suitable r shiny app (Schoemann et al., 2017) for a model with three parallel mediators with a target power of 1 – β = .80, 1000 replications, 95% confidence levels and a correlation matrix comprising low-to-moderate effect sizes (r = .25) showed that a sample of N = 380 is needed to detect indirect effects.

**Table S1**

*Means, standard deviations, and correlations with confidence intervals*

| Variable | *M* | *SD* | 1 | 2 | 3 | 4 | 5 | 6 | 7 | 8 | 9 |
| --- | --- | --- | --- | --- | --- | --- | --- | --- | --- | --- | --- |
|  |  |  |  |  |  |  |  |  |  |  |  |
| 1. Jewish identification | 4.59 | 1.03 |  |  |  |  |  |  |  |  |  |
|  |  |  |  |  |  |  |  |  |  |  |  |
| 2. Vigilance | 2.95 | 0.89 | -.00 |  |  |  |  |  |  |  |  |
|  |  |  | [-.11, .10] |  |  |  |  |  |  |  |  |
|  |  |  |  |  |  |  |  |  |  |  |  |
| 3. Perceived AS | 45.00 | 21.37 | .20** | .25** |  |  |  |  |  |  |  |
|  |  |  | [.10, .29] | [.15, .35] |  |  |  |  |  |  |  |
|  |  |  |  |  |  |  |  |  |  |  |  |
| 4. Everyday AS | 1.66 | 0.62 | .14** | .27** | .27** |  |  |  |  |  |  |
|  |  |  | [.03, .24] | [.17, .36] | [.17, .36] |  |  |  |  |  |  |
|  |  |  |  |  |  |  |  |  |  |  |  |
| 5. Subtle AS | 2.93 | 1.01 | .30** | .30** | .34** | .60** |  |  |  |  |  |
|  |  |  | [.20, .39] | [.20, .40] | [.25, .43] | [.53, .67] |  |  |  |  |  |
|  |  |  |  |  |  |  |  |  |  |  |  |
| 6. Collective AS | 3.36 | 0.91 | .37** | .41** | .33** | .44** | .67** |  |  |  |  |
|  |  |  | [.28, .45] | [.32, .49] | [.24, .42] | [.35, .52] | [.61, .72] |  |  |  |  |
|  |  |  |  |  |  |  |  |  |  |  |  |
| 7. SWB | 4.59 | 1.16 | .10 | -.08 | -.17** | -.30** | -.09 | -.08 |  |  |  |
|  |  |  | [-.00, .20] | [-.18, .03] | [-.27, -.07] | [-.39, -.20] | [-.20, .01] | [-.18, .02] |  |  |  |
|  |  |  |  |  |  |  |  |  |  |  |  |
| 8. Anxiety | 1.99 | 0.56 | -.02 | .16** | .11* | .38** | .33** | .27** | -.45** |  |  |
|  |  |  | [-.12, .08] | [.06, .26] | [.00, .21] | [.28, .46] | [.23, .42] | [.17, .36] | [-.53, -.37] |  |  |
|  |  |  |  |  |  |  |  |  |  |  |  |
| 9. Depression | 1.64 | 0.47 | -.02 | .14** | .21** | .35** | .17** | .15** | -.59** | .54** |  |
|  |  |  | [-.12, .08] | [.04, .24] | [.11, .31] | [.25, .43] | [.07, .27] | [.05, .25] | [-.65, -.52] | [.46, .60] |  |
|  |  |  |  |  |  |  |  |  |  |  |  |
| 10. Social participation | 3.16 | 0.73 | .02 | -.19** | -.12* | -.19** | -.08 | -.07 | .38** | -.26** | -.44** |
|  |  |  | [-.09, .12] | [-.29, -.09] | [-.22, -.01] | [-.29, -.09] | [-.18, .02] | [-.17, .03] | [.29, .47] | [-.35, -.16] | [-.52, -.35] |
|  |  |  |  |  |  |  |  |  |  |  |  |

*Note.* AS = Antisemitism. SWB = Subjective well-being. *M* and *SD* are used to represent mean and standard deviation, respectively. Values in square brackets indicate the 95% confidence interval for each correlation. * indicates *p* < 0.05. ** indicates *p* < 0.01.

**Table S2**

*Linear Regression Predicting Subjective Well-Being*

|  | **Subjective Well-Being** | | | | | | |
| --- | --- | --- | --- | --- | --- | --- | --- |
| *Predictors* | *B* | *std. Error* | *std. Beta* | *standardized std. Error* | *95% CI* | *standardized CI* | *p* |
| Intercept | 2.554 | 0.455 | -0.000 | 0.047 | 1.660 – 3.449 | -0.092 – 0.092 | **<0.001** |
| Age | 0.001 | 0.001 | 0.105 | 0.048 | 0.000 – 0.002 | 0.011 – 0.200 | **0.030** |
| 1 = female | -0.127 | 0.112 | -0.054 | 0.048 | -0.347 – 0.094 | -0.149 – 0.040 | 0.260 |
| 1 = migration background | 0.073 | 0.143 | 0.026 | 0.050 | -0.208 – 0.355 | -0.073 – 0.124 | 0.608 |
| 1 = In relationship | 0.143 | 0.123 | 0.061 | 0.053 | -0.100 – 0.386 | -0.043 – 0.164 | 0.249 |
| No. of children | 0.041 | 0.054 | 0.040 | 0.052 | -0.064 – 0.147 | -0.062 – 0.143 | 0.441 |
| Religiosity | -0.008 | 0.027 | -0.019 | 0.061 | -0.061 – 0.045 | -0.139 – 0.102 | 0.763 |
| Socioeconomic Status | 0.521 | 0.073 | 0.357 | 0.050 | 0.377 – 0.665 | 0.258 – 0.456 | **<0.001** |
| Right-wing orientation | 0.006 | 0.031 | 0.010 | 0.049 | -0.054 – 0.066 | -0.088 – 0.107 | 0.846 |
| Time spent in the Jewish community | 0.066 | 0.056 | 0.067 | 0.057 | -0.045 – 0.177 | -0.045 – 0.180 | 0.242 |
| Jewish identification | 0.085 | 0.064 | 0.076 | 0.058 | -0.041 – 0.212 | -0.037 – 0.190 | 0.186 |
| Vigilance | 0.048 | 0.070 | 0.038 | 0.055 | -0.090 – 0.186 | -0.070 – 0.145 | 0.494 |
| Perceived Antisemitism | -0.006 | 0.003 | -0.121 | 0.053 | -0.012 – -0.001 | -0.226 – -0.017 | **0.023** |
| Everyday Antisemitism | -0.554 | 0.115 | -0.303 | 0.063 | -0.781 – -0.328 | -0.427 – -0.179 | **<0.001** |
| Subtle Antisemitism | 0.161 | 0.086 | 0.141 | 0.075 | -0.008 – 0.330 | -0.007 – 0.288 | 0.061 |
| Collective Antisemitism | 0.004 | 0.089 | 0.003 | 0.071 | -0.170 – 0.178 | -0.136 – 0.142 | 0.964 |
| Observations | 332 | | | | | | |
| R^2^ / R^2^ adjusted | 0.303 / 0.269 | | | | | | |

**Table S3**

*Linear Regression Predicting Anxiety*

|  | **Anxiety** | | | | | | |
| --- | --- | --- | --- | --- | --- | --- | --- |
| *Predictors* | *B* | *std. Error* | *std. Beta* | *standardized std. Error* | *95% CI* | *standardized CI* | *p* |
| Intercept | 1.748 | 0.228 | 0.000 | 0.049 | 1.301 – 2.196 | -0.097 – 0.097 | **<0.001** |
| Age | -0.000 | 0.000 | -0.000 | 0.051 | -0.001 – 0.001 | -0.100 – 0.099 | 0.995 |
| 1 = female | 0.165 | 0.056 | 0.149 | 0.051 | 0.055 – 0.276 | 0.050 – 0.249 | **0.003** |
| 1 = migration background | 0.003 | 0.072 | 0.002 | 0.053 | -0.138 – 0.144 | -0.101 – 0.105 | 0.968 |
| 1 = In relationship | 0.022 | 0.062 | 0.020 | 0.055 | -0.099 – 0.144 | -0.089 – 0.129 | 0.718 |
| No. of children | -0.050 | 0.027 | -0.102 | 0.055 | -0.103 – 0.003 | -0.210 – 0.006 | 0.063 |
| Religiosity | -0.015 | 0.013 | -0.071 | 0.064 | -0.041 – 0.012 | -0.197 – 0.056 | 0.274 |
| Socioeconomic Status | -0.066 | 0.037 | -0.095 | 0.053 | -0.138 – 0.006 | -0.198 – 0.009 | 0.073 |
| Right-wing orientation | -0.033 | 0.015 | -0.112 | 0.052 | -0.063 – -0.003 | -0.214 – -0.010 | **0.032** |
| Time spent in the Jewish community | 0.003 | 0.028 | 0.006 | 0.060 | -0.053 – 0.058 | -0.112 – 0.125 | 0.918 |
| Jewish identification | -0.035 | 0.032 | -0.065 | 0.061 | -0.098 – 0.029 | -0.185 – 0.054 | 0.283 |
| Vigilance | 0.005 | 0.035 | 0.009 | 0.058 | -0.064 – 0.074 | -0.105 – 0.122 | 0.879 |
| Perceived Antisemitism | -0.000 | 0.001 | -0.013 | 0.056 | -0.003 – 0.002 | -0.123 – 0.096 | 0.812 |
| Everyday Antisemitism | 0.271 | 0.058 | 0.311 | 0.066 | 0.158 – 0.385 | 0.181 – 0.441 | **<0.001** |
| Subtle Antisemitism | 0.027 | 0.043 | 0.049 | 0.079 | -0.057 – 0.111 | -0.105 – 0.204 | 0.529 |
| Collective Antisemitism | 0.069 | 0.044 | 0.116 | 0.074 | -0.018 – 0.156 | -0.030 – 0.262 | 0.120 |
| Observations | 333 | | | | | | |
| R^2^ / R^2^ adjusted | 0.229 / 0.192 | | | | | | |

**Table S4**

*Linear Regression Predicting Depression*

|  | **Depression** | | | | | | |
| --- | --- | --- | --- | --- | --- | --- | --- |
| *Predictors* | *B* | *std. Error* | *std. Beta* | *standardized std. Error* | *95% CI* | *standardized CI* | *p* |
| Intercept | 1.679 | 0.194 | 0.000 | 0.050 | 1.297 – 2.061 | -0.099 – 0.099 | **<0.001** |
| Age | -0.000 | 0.000 | -0.027 | 0.052 | -0.001 – 0.000 | -0.128 – 0.075 | 0.603 |
| 1 = female | 0.002 | 0.048 | 0.003 | 0.052 | -0.092 – 0.097 | -0.099 – 0.104 | 0.958 |
| 1 = migration background | -0.078 | 0.061 | -0.068 | 0.054 | -0.198 – 0.042 | -0.173 – 0.037 | 0.204 |
| 1 = In relationship | 0.003 | 0.053 | 0.003 | 0.056 | -0.101 – 0.107 | -0.108 – 0.114 | 0.957 |
| No. of children | 0.008 | 0.023 | 0.020 | 0.056 | -0.037 – 0.053 | -0.090 – 0.130 | 0.719 |
| Religiosity | -0.003 | 0.012 | -0.014 | 0.066 | -0.025 – 0.020 | -0.144 – 0.115 | 0.829 |
| Socioeconomic Status | -0.096 | 0.031 | -0.164 | 0.054 | -0.157 – -0.034 | -0.270 – -0.058 | **0.002** |
| Right-wing orientation | 0.008 | 0.013 | 0.031 | 0.053 | -0.018 – 0.033 | -0.073 – 0.135 | 0.556 |
| Time spent in the Jewish community | -0.035 | 0.024 | -0.090 | 0.061 | -0.083 – 0.012 | -0.211 – 0.030 | 0.142 |
| Jewish identification | -0.016 | 0.028 | -0.036 | 0.062 | -0.070 – 0.039 | -0.158 – 0.087 | 0.566 |
| Vigilance | 0.006 | 0.030 | 0.012 | 0.059 | -0.053 – 0.065 | -0.103 – 0.128 | 0.836 |
| Perceived Antisemitism | 0.003 | 0.001 | 0.128 | 0.057 | 0.000 – 0.005 | 0.016 – 0.240 | **0.025** |
| Everyday Antisemitism | 0.267 | 0.049 | 0.366 | 0.068 | 0.170 – 0.364 | 0.233 – 0.499 | **<0.001** |
| Subtle Antisemitism | -0.071 | 0.037 | -0.156 | 0.080 | -0.144 – 0.001 | -0.314 – 0.002 | 0.053 |
| Collective Antisemitism | 0.039 | 0.038 | 0.078 | 0.076 | -0.036 – 0.113 | -0.071 – 0.227 | 0.305 |
| Observations | 332 | | | | | | |
| R^2^ / R^2^ adjusted | 0.201 / 0.163 | | | | | | |

**Table S5**

*Linear Regression Predicting social participation*

|  | **Social Participation** | | | | | | |
| --- | --- | --- | --- | --- | --- | --- | --- |
| *Predictors* | *B* | *std. Error* | *std. Beta* | *standardized std. Error* | *95% CI* | *standardized CI* | *p* |
| Intercept | 2.869 | 0.317 | 0.000 | 0.051 | 2.245 – 3.492 | -0.100 – 0.100 | **<0.001** |
| Age | -0.000 | 0.000 | -0.008 | 0.052 | -0.001 – 0.001 | -0.112 – 0.095 | 0.872 |
| 1 = female | 0.066 | 0.078 | 0.044 | 0.053 | -0.088 – 0.220 | -0.059 – 0.147 | 0.401 |
| 1 = migration background | 0.149 | 0.100 | 0.081 | 0.054 | -0.047 – 0.345 | -0.026 – 0.189 | 0.136 |
| 1 = In relationship | -0.019 | 0.087 | -0.012 | 0.058 | -0.189 – 0.152 | -0.126 – 0.101 | 0.828 |
| No. of children | -0.122 | 0.038 | -0.185 | 0.057 | -0.196 – -0.047 | -0.298 – -0.072 | **0.001** |
| Religiosity | -0.038 | 0.019 | -0.137 | 0.067 | -0.075 – -0.001 | -0.269 – -0.005 | **0.041** |
| Socioeconomic Status | 0.128 | 0.051 | 0.136 | 0.055 | 0.027 – 0.228 | 0.029 – 0.243 | **0.013** |
| Right-wing orientation | -0.005 | 0.021 | -0.014 | 0.054 | -0.047 – 0.037 | -0.120 – 0.092 | 0.800 |
| Time spent in the Jewish community | 0.173 | 0.039 | 0.273 | 0.062 | 0.095 – 0.250 | 0.151 – 0.396 | **<0.001** |
| Jewish identification | -0.012 | 0.045 | -0.017 | 0.063 | -0.100 – 0.076 | -0.141 – 0.107 | 0.786 |
| Vigilance | -0.107 | 0.049 | -0.129 | 0.060 | -0.204 – -0.010 | -0.247 – -0.012 | **0.031** |
| Perceived Antisemitism | -0.002 | 0.002 | -0.068 | 0.058 | -0.006 – 0.002 | -0.181 – 0.045 | 0.239 |
| Everyday Antisemitism | -0.211 | 0.080 | -0.180 | 0.068 | -0.369 – -0.053 | -0.314 – -0.045 | **0.009** |
| Subtle Antisemitism | 0.111 | 0.060 | 0.151 | 0.081 | -0.007 – 0.229 | -0.009 – 0.310 | 0.064 |
| Collective Antisemitism | -0.015 | 0.062 | -0.019 | 0.077 | -0.137 – 0.107 | -0.169 – 0.132 | 0.809 |
| Observations | 331 | | | | | | |
| R^2^ / R^2^ adjusted | 0.176 / 0.137 | | | | | | |

**Table S6**

*Linear Regressions Predicting Outcome Variables with Interaction Terms*

|  | **Subjective Well-Being** | | | **Anxiety** | | | **Depression** | | | **Social Participation** | | |
| --- | --- | --- | --- | --- | --- | --- | --- | --- | --- | --- | --- | --- |
| *Predictors* | *B* | *95% CI* | *p* | *B* | *95% CI* | *p* | *B* | *95% CI* | *p* | *B* | *95% CI* | *p* |
| Intercept | 2.141 | 1.433 – 2.849 | **<0.001** | 2.322 | 1.970 – 2.673 | **<0.001** | 2.136 | 1.827 – 2.446 | **<0.001** | 2.295 | 1.809 – 2.780 | **<0.001** |
| Age | 0.001 | -0.000 – 0.002 | 0.072 | -0.000 | -0.001 – 0.000 | 0.882 | -0.000 | -0.000 – 0.000 | 0.879 | -0.000 | -0.001 – 0.001 | 0.834 |
| 1 = female | -0.156 | -0.371 – 0.059 | 0.155 | 0.177 | 0.070 – 0.283 | **0.001** | 0.010 | -0.084 – 0.104 | 0.836 | 0.040 | -0.108 – 0.188 | 0.596 |
| 1 = migration background | 0.100 | -0.174 – 0.375 | 0.474 | 0.027 | -0.109 – 0.164 | 0.692 | -0.045 | -0.165 – 0.075 | 0.461 | 0.100 | -0.088 – 0.288 | 0.297 |
| 1 = In relationship | 0.199 | -0.033 – 0.432 | 0.093 | 0.010 | -0.106 – 0.125 | 0.869 | -0.032 | -0.134 – 0.069 | 0.536 | -0.008 | -0.169 – 0.152 | 0.920 |
| No. of children | 0.048 | -0.052 – 0.149 | 0.345 | -0.051 | -0.101 – -0.001 | **0.046** | 0.010 | -0.033 – 0.054 | 0.639 | -0.106 | -0.175 – -0.036 | **0.003** |
| Religiosity | 0.008 | -0.043 – 0.060 | 0.748 | -0.009 | -0.034 – 0.017 | 0.506 | -0.003 | -0.025 – 0.020 | 0.806 | -0.039 | -0.074 – -0.003 | **0.032** |
| Socioeconomic Status | 0.561 | 0.421 – 0.702 | **<0.001** | -0.067 | -0.137 – 0.002 | 0.058 | -0.116 | -0.178 – -0.055 | **<0.001** | 0.146 | 0.050 – 0.243 | **0.003** |
| Right-wing orientation | -0.003 | -0.061 – 0.055 | 0.912 | -0.037 | -0.065 – -0.008 | **0.012** | 0.014 | -0.011 – 0.040 | 0.270 | -0.011 | -0.050 – 0.029 | 0.600 |
| Time spent in the Jewish community | 0.060 | -0.049 – 0.168 | 0.281 | 0.006 | -0.048 – 0.060 | 0.819 | -0.028 | -0.075 – 0.020 | 0.249 | 0.178 | 0.103 – 0.252 | **<0.001** |
| Jewish identification | 0.078 | -0.037 – 0.193 | 0.183 | -0.013 | -0.070 – 0.044 | 0.653 | -0.020 | -0.071 – 0.031 | 0.438 | -0.011 | -0.090 – 0.068 | 0.789 |
| Everyday Antisemitism | -0.436 | -0.617 – -0.255 | **<0.001** | 0.333 | 0.243 – 0.423 | **<0.001** | 0.245 | 0.166 – 0.324 | **<0.001** | -0.164 | -0.288 – -0.041 | **0.009** |
| Identification X Everyday Antisemitism | -0.021 | -0.209 – 0.166 | 0.822 | 0.020 | -0.073 – 0.113 | 0.676 | -0.006 | -0.088 – 0.076 | 0.886 | -0.067 | -0.196 – 0.062 | 0.306 |
| Observations | 352 | | | 353 | | | 352 | | | 351 | | |
| R^2^ / R^2^ adjusted | 0.286 / 0.261 | | | 0.220 / 0.192 | | | 0.175 / 0.146 | | | 0.147 / 0.117 | | |

**Table S7**

*Means (SDs) and Results of One-Way ANOVAs for Differences Between Profiles in Profiling Variables*

|  | Profile 1: High identity, high antisemitism (*n* = 181) | Profile 2: Low identity, low antisemitism (*n* = 29) | Profile 3: Average identity, low antisemitism (*n* = 134) | ANOVA | Significance |  |  |
| --- | --- | --- | --- | --- | --- | --- | --- |
|  |  |  |  |  | Profile 2 vs. Profile 1 | Profile 3 vs. profile 1 | Profile 3 vs. profile 2 |
|  |  |  |  |  |  |  |  |
| Jewish identification | 4.96 (0.73) | 2.38 (0.69) | 4.57 (0.83) | F(2,341) = 142.57, p < .001 | *** | *** | *** |
| Vigilance | 3.06 (0.87) | 2.74 (0.98) | 2.85 (0.90) | F(2,341) = 2.97, p = .06 |  |  |  |
| Perceived AS | 47.97 (21.63) | 41.28 (23.47) | 41.90 (20.12) | F(2,341) = 3.64, p = .03 |  |  |  |
| Everyday AS | 1.96 (0.66) | 1.42 (0.39) | 1.36 (0.40) | F(2,341) = 49.76, p < .001 | *** | *** |  |
| Subtle AS | 3.69 (0.65) | 2.57 (0.79) | 2.06 (0.55) | F(2,341) = 268.88, p < .001 | *** | *** | *** |
| Collective AS | 3.94 (0.63) | 2.67 (0.79) | 2.81 (0.77) | F(2,341) = 115.13, p < .001 | *** | *** |  |

*Note*. Values are unstandardized. AS = Antisemitism. Range for perceived antisemitism: 1-90, Jewish identification: 1-6, all other variables: 1-5. Pairwise comparisons are with Tukey adjustment.

* *p* < .05. ** *p* < .01. *** *p* < .001.

**Table S8**

*Demographic Information for the Three profiles with Significance Tests for the Difference Between Profiles*

| Variable | Profile | | | Test |  |
| --- | --- | --- | --- | --- | --- |
|  | Profile 1: High identity, high antisemitism (*n* = 181) | Profile 2: Low identity, low antisemitism (*n* = 29) | Profile 3: Average identity, low antisemitism (*n* = 134) |  |  |
| Age | *M* (*SD*) | *M* (*SD*) | *M* (*SD*) | ANOVA/ χ^2^ |  |
|  | 38.68 (14.55) | 35.10 (11.92) | 42.23 (17.42) | *F*(2,341) = 3.43, *p* = .03, η^2^ = .02 |  |
| Gender | *n* (%) | *n* (%) | *n* (%) | χ^2^ (2) = 1.11, *p* = .57 |  |
| Male | 75 (42%) | 14 (48%) | 51 (38%) |  |  |
| Female | 103 (58%) | 15 (52%) | 82 (62%) |  |  |
| Coutnry of birth |  |  |  | χ^2^ (6) = 22.02, *p* = .011 |  |
| Germany | 112 (62%)^a,b^ | 10 (36%)^a^ | 57 (43%)^b^ |  |  |
| FSU | 38 (21%)^a,b^ | 8 (29%)^a^ | 46 (34%)^b^ |  |  |
| Israel | 14 (8%)^a^ | 8 (29%)^a,b^ | 18 (13%)^b^ |  |  |
| Other | 16 (9%) | 2 (7%) | 13 (10%) |  |  |
| Socio-economic status | 3.72 (0.76) | 3.46 (0.64) | 3.82 (0.83) | *F*(2,338) = 2.54, *p* = .08 |  |
| Political orientation | 4.46 (1.86) | 4.07 (2.12) | 4.42 (1.84) | *F*(2,336) = 0.51, *p* = .60 |  |
| Religiosity | 5.12 (2.43)^a^ | 2.00 (2.41)^a^ | 3.95 (2.54)^a^ | *F*(2,341) = 23.66, *p* < .001, η^2^ = .12 |  |
| Time spent in the Jewish community | 3.27 (1.15)^a^ | 2.10 (0.98)^a^ | 2.87 (1.12)^a^ | *F*(2,341) = 15.02, *p* < .001, η^2^ = .08 |  |
| Family Status |  |  |  | χ^2^ (2) = 1.40, *p* = .50 |  |
| Not in a relationship | 68 (38%) | 11 (38%) | 59 (44%) |  |  |
| In a relationship | 113 (62%) | 18 (62%) | 75 (56%) |  |  |
| Number of children | 0.97 (1.16) | 0.62 (0.82) | 0.95 (1.13) | *F*(2,341) = 1.25, *p* = .29 |  |

*Note*. *N* = 339-344. FSU = Former Soviet Union. Identical row letters denote significant difference between profiles based on Bonferroni-corrected pairwise comparisons (*p* < .05).

**Table S9**

*Model Fit Indices of Latent Profile Analysis.*

|  | Model | Classes | LogLik | AIC | AWE | BIC | CAIC | CLC | KIC | SABIC | ICL | Entropy | prob_min | prob_max | n_min | n_max | BLRT_val | BLRT_p |
| --- | --- | --- | --- | --- | --- | --- | --- | --- | --- | --- | --- | --- | --- | --- | --- | --- | --- | --- |
| 1 | 1 | 1 | -2920.16 | 5864.324 | 6014.499 | 5910.411 | 5922.411 | 5842.324 | 5879.324 | 5872.344 | -5910.41 | 1 | 1 | 1 | 1 | 1 | NA | NA |
| 2 | 1 | 2 | -2727.34 | 5492.67 | 5732.066 | 5565.642 | 5584.642 | 5456.219 | 5514.67 | 5505.369 | -5619.15 | 0.774464 | 0.933319 | 0.935016 | 0.476744 | 0.523256 | 385.6536 | 0.009901 |
| 3 | 1 | 3 | -2659.88 | 5371.764 | 5699.925 | 5471.62 | 5497.62 | 5321.316 | 5400.764 | 5389.142 | -5553.62 | 0.775919 | 0.892221 | 0.909053 | 0.261628 | 0.468023 | 134.9063 | 0.009901 |
| 4 | 1 | 4 | -2630.44 | 5326.884 | 5743.744 | 5453.625 | 5486.625 | 5262.506 | 5362.884 | 5348.941 | -5544.53 | 0.810946 | 0.879539 | 0.9396 | 0.020349 | 0.459302 | 58.87994 | 0.009901 |
| 5 | 1 | 5 | -2635.27 | 5350.531 | 5856.336 | 5504.157 | 5544.157 | 5271.978 | 5393.531 | 5377.267 | -5654.24 | 0.723389 | 0.612615 | 0.914353 | 0.061047 | 0.299419 | -9.64741 | 0.524752 |
| 6 | 1 | 6 | -2600.05 | 5294.094 | 5888.567 | 5474.605 | 5521.605 | 5201.643 | 5344.094 | 5325.509 | -5610.14 | 0.774083 | 0.780383 | 0.895341 | 0.017442 | 0.34593 | 70.43683 | 0.009901 |
| 7 | 1 | 7 | -2578.13 | 5264.251 | 5947.372 | 5471.646 | 5525.646 | 5157.919 | 5321.251 | 5300.344 | -5576.58 | 0.834047 | 0.813649 | 0.93041 | 0.052326 | 0.340116 | 43.84337 | 0.009901 |
| 8 | 1 | 8 | -2580.6 | 5283.202 | 6055.185 | 5517.481 | 5578.481 | 5162.777 | 5347.202 | 5323.973 | -5665.42 | 0.787483 | 0.754405 | 0.892233 | 0.037791 | 0.212209 | -4.95093 | 0.653465 |
| 9 | 1 | 9 | -2552.72 | 5241.447 | 6102.17 | 5502.611 | 5570.611 | 5107.051 | 5312.447 | 5286.897 | -5646.25 | 0.801926 | 0.719554 | 0.978047 | 0.020349 | 0.241279 | 55.7551 | 0.009901 |
| 10 | 1 | 10 | -2537.29 | 5224.577 | 6174.038 | 5512.625 | 5587.625 | 5076.212 | 5302.577 | 5274.706 | -5650.05 | 0.817735 | 0.754073 | 0.988044 | 0.020349 | 0.18314 | 30.86992 | 0.009901 |
| 11 | 2 | 1 | -2920.16 | 5864.324 | 6014.499 | 5910.411 | 5922.411 | 5842.324 | 5879.324 | 5872.344 | -5910.41 | 1 | 1 | 1 | 1 | 1 | NA | NA |
| 12 | 2 | 2 | -2682.91 | 5415.827 | 5731.283 | 5511.844 | 5536.844 | 5367.404 | 5443.827 | 5432.537 | -5562.61 | 0.788051 | 0.936037 | 0.939507 | 0.47093 | 0.52907 | 474.4962 | 0.009901 |
| 13 | 2 | 3 | -2600.39 | 5276.786 | 5757.064 | 5422.73 | 5460.73 | 5202.397 | 5317.786 | 5302.185 | -5496.61 | 0.805618 | 0.901593 | 0.91559 | 0.186047 | 0.520349 | 165.0414 | 0.009901 |
| 14 | 2 | 4 | -2565.74 | 5233.484 | 5878.532 | 5429.357 | 5480.357 | 5133.181 | 5287.484 | 5267.571 | -5498.1 | 0.848704 | 0.90067 | 0.920396 | 0.156977 | 0.377907 | 69.30224 | 0.009901 |
| 15 | 2 | 5 | -2553.11 | 5234.21 | 6044.192 | 5480.011 | 5544.011 | 5107.831 | 5301.21 | 5276.987 | -5584.84 | 0.81052 | 0.776506 | 0.912316 | 0.104651 | 0.299419 | 25.2734 | 0.346535 |
| 16 | 2 | 6 | -2511.09 | 5176.186 | 6151.032 | 5471.916 | 5548.916 | 5023.799 | 5256.186 | 5227.652 | -5584.68 | 0.806535 | 0.699321 | 0.964158 | 0.078488 | 0.276163 | 84.02399 | 0.009901 |
| 17 | 2 | 7 | -2496.93 | 5173.866 | 6313.599 | 5519.524 | 5609.524 | 4995.449 | 5266.866 | 5234.021 | -5653.08 | 0.79151 | 0.766139 | 0.934296 | 0.078488 | 0.218023 | 28.32032 | 0.237624 |
| 18 | 2 | 8 | -2463.73 | 5133.456 | 6438.007 | 5529.042 | 5632.042 | 4929.076 | 5239.456 | 5202.299 | -5656.09 | 0.81016 | 0.745589 | 0.943838 | 0.063953 | 0.273256 | 66.41049 | 0.009901 |
| 19 | 2 | 9 | -2448.05 | 5128.094 | 6597.479 | 5573.608 | 5689.608 | 4897.738 | 5247.094 | 5205.627 | -5696.24 | 0.822106 | 0.757012 | 0.946858 | 0.05814 | 0.186047 | 31.36165 | 0.059406 |
| 20 | 2 | 10 | NA | NA | NA | NA | NA | NA | NA | NA | NA | NA | NA | NA | NA | NA | NA | NA |
| 21 | 3 | 1 | -2636.84 | 5327.689 | 5668.084 | 5431.387 | 5458.387 | 5275.689 | 5357.689 | 5345.736 | -5431.39 | 1 | 1 | 1 | 1 | 1 | NA | NA |
| 22 | 3 | 2 | -2622.78 | 5313.558 | 5743.334 | 5444.14 | 5478.14 | 5246.946 | 5350.558 | 5336.283 | -5512.91 | 0.693836 | 0.886956 | 0.934094 | 0.40407 | 0.59593 | 28.13087 | 0.019802 |
| 23 | 3 | 3 | -2607.04 | 5296.077 | 5814.642 | 5453.543 | 5494.543 | 5215.445 | 5340.077 | 5323.481 | -5572.32 | 0.683776 | 0.721503 | 0.901694 | 0.084302 | 0.526163 | 31.48117 | 0.009901 |
| 24 | 3 | 4 | -2563.66 | 5223.322 | 5830.227 | 5407.673 | 5455.673 | 5129.119 | 5274.322 | 5255.405 | -5445.71 | 0.89836 | 0.763936 | 0.974621 | 0.014535 | 0.613372 | 86.75507 | 0.009901 |
| 25 | 3 | 5 | -2569.93 | 5249.862 | 5945.948 | 5461.097 | 5516.097 | 5141.246 | 5307.862 | 5286.623 | -5627.69 | 0.69211 | 0.677944 | 0.862282 | 0.061047 | 0.363372 | -12.54 | 0.910891 |
| 26 | 3 | 6 | -2566.99 | 5257.98 | 6042.855 | 5496.1 | 5558.1 | 5135.345 | 5322.98 | 5299.42 | -5684.47 | 0.682499 | 0.552965 | 0.843462 | 0.06686 | 0.366279 | 5.881654 | 0.910891 |
| 27 | 3 | 7 | -2532.82 | 5203.642 | 6077.016 | 5468.646 | 5537.646 | 5067.276 | 5275.642 | 5249.76 | -5575.89 | 0.817158 | 0.77036 | 0.891488 | 0.026163 | 0.319767 | 68.33867 | 0.009901 |
| 28 | 3 | 8 | -2525.95 | 5203.904 | 6166.091 | 5495.793 | 5571.793 | 5053.495 | 5282.904 | 5254.701 | -5629.45 | 0.795304 | 0.709668 | 0.899558 | 0.026163 | 0.270349 | 13.73767 | 0.316832 |
| 29 | 3 | 9 | -2516.95 | 5199.896 | 6250.899 | 5518.669 | 5601.669 | 5035.439 | 5285.896 | 5255.372 | -5682.44 | 0.771789 | 0.685504 | 0.970744 | 0.014535 | 0.226744 | 18.00824 | 0.049505 |
| 30 | 3 | 10 | -2513.25 | 5206.508 | 6346.301 | 5552.166 | 5642.166 | 5028.03 | 5299.508 | 5266.662 | -5735.68 | 0.761086 | 0.575629 | 0.974365 | 0.014535 | 0.229651 | 7.388022 | 0.782178 |
| 31 | 6 | 1 | -2636.84 | 5327.689 | 5668.084 | 5431.387 | 5458.387 | 5275.689 | 5357.689 | 5345.736 | -5431.39 | 1 | 1 | 1 | 1 | 1 | NA | NA |
| 32 | 6 | 2 | -2566.38 | 5242.764 | 5938.923 | 5454 | 5509 | 5134.077 | 5300.764 | 5279.525 | -5541.83 | 0.656103 | 0.880924 | 0.903123 | 0.418605 | 0.581395 | 140.9249 | 0.009901 |
| 33 | 6 | 3 | -2518.12 | 5202.24 | 6253.228 | 5521.013 | 5604.013 | 5037.799 | 5288.24 | 5257.716 | -5595.33 | 0.779384 | 0.880412 | 0.923404 | 0.247093 | 0.473837 | 96.52426 | 0.009901 |
| 34 | 6 | 4 | -2507.98 | 5237.95 | 6643.959 | 5664.261 | 5775.261 | 5017.563 | 5351.95 | 5312.141 | -5752.14 | 0.806609 | 0.866044 | 0.944283 | 0.127907 | 0.386628 | 20.28997 | 0.980198 |
| 35 | 6 | 5 | -2469.94 | 5217.874 | 6978.953 | 5751.723 | 5890.723 | 4941.492 | 5359.874 | 5310.779 | -5853.04 | 0.809359 | 0.784285 | 0.960522 | 0.104651 | 0.343023 | 76.07629 | 0.059406 |
| 36 | 6 | 6 | -2419.6 | 5173.21 | 7289.289 | 5814.597 | 5981.597 | 4840.904 | 5343.21 | 5284.83 | -5899.94 | 0.847351 | 0.833061 | 0.970643 | 0.098837 | 0.27907 | 100.6641 | 0.009901 |
| 37 | 6 | 7 | -2399.12 | 5188.249 | 7659.378 | 5937.174 | 6132.174 | 4799.969 | 5386.249 | 5318.584 | -6021.22 | 0.860335 | 0.85961 | 0.999334 | 0.034884 | 0.293605 | 40.96105 | 0.148515 |
| 38 | 6 | 8 | -2343.35 | 5132.709 | 7958.917 | 5989.172 | 6212.172 | 4688.427 | 5358.709 | 5281.759 | -6080.55 | 0.859093 | 0.81274 | 0.982531 | 0.075581 | 0.238372 | 111.5397 | 0.009901 |
| 39 | 6 | 9 | -2321.13 | 5144.25 | 8325.491 | 6108.251 | 6359.251 | 4644.012 | 5398.25 | 5312.015 | -6186.44 | 0.880768 | 0.780723 | 0.992156 | 0.040698 | 0.223837 | 44.45854 | 0.633663 |
| 40 | 6 | 10 | -2293.38 | 5144.754 | 8681.008 | 6216.293 | 6495.293 | 4588.578 | 5426.754 | 5331.233 | -6271.85 | 0.911931 | 0.871265 | 0.991541 | 0.034884 | 0.171512 | 55.4966 | 0.168317 |

*Note*. The selected model is highlighted.
